# Controllable *in-situ* cell electroporation with cell positioning and impedance monitoring using micro electrode array

**DOI:** 10.1038/srep31392

**Published:** 2016-08-10

**Authors:** Xiaoliang Guo, Rong Zhu

**Affiliations:** 1 State Key Laboratory of Precision Measurement Technology and Instruments, Department of Precision Instrument, Tsinghua University, Beijing, 100084, China

## Abstract

This paper reports a novel microarray chip for *in-situ*, real-time and selective electroporation on individual cells integrated with cell positioning and impedance monitoring. An array of quadrupole-electrode units (termed positioning electrodes) and pairs of planar center electrodes located at the centers of each quadrupole-electrode unit were fabricated on the chip. The positioning electrodes are used to trap and position living cells onto the center electrodes based on negative dielectrophoresis (nDEP). The center electrodes are used for *in-situ* cell electroporation, and also used to measure cell impedance for monitoring cellular dynamics in real time. Controllably selective electroporation and electrical measurement on the cells in array are realized. We present an evidence of selective electroporation through use of fluorescent dyes. Subsequently we use *in-situ* and real-time impedance measurement to monitor the process, which demonstrates the dynamic behavior of the cell electroporation. Finally, we show the use of this device to perform successful transfection onto individual HeLa cells with vector DNA encoding a green fluorescent.

Delivering nucleic acids into eukaryotic cells, a process known as transfection, has enabled a wide range of applications including gene therapy, DNA vaccines, *in vitro* fertilization, cancer treatment, regenerative medicine and induced pluripotent stem (iPS) cells^1^. Viral methods (e.g. viral vector)^2^, and chemical method (e.g. calcium phosphate method)^3^ are commonly used for transfection. Viral method remains a high efficient means by which exogenous genes can be introduced into and expressed by mammalian cells. Retrovirus, adenovirus, adeno-associated virus and herpes virus are widely studied by viral gene transfer systems and have attracted the most attentions in the field of transfection^2^. Chemical methods make use of reagents such as cationic lipids or polymers complexed with DNA to transfer^2^. However, they have some drawbacks, such as immune responses, unwanted mutagenesis, toxicity, and the possibility of developing cancer^4^. Physical method provides an alternative gene transfection approach which is safe, label-free, simple and able to transfect large DNA^5^. Physical methods including microinjection^6^, ultrasound^7^, laser irradiation^8^,^9^ and electroporation generate holes in the plasma membrane for direct delivery of exogenous molecules into the cytoplasm. Among the physical methods, electroporation is simple and can be applied to various cell types^10^ and has become one of the mainstreams of transfection. Electroporation is a phenomenon related with a state of increased permeability of different exogenous molecules into the plasma membrane of biological structures when high electric field pulses are applied to the cells. The electroporation has now been commercially used. However, most electroporation machines are bulky and high voltages (hundreds or even thousands of volts) are generally required to generate sufficient electrical fields for efficient electroporation. The process may cause cell damaged and unexpected risks in operation^1^. In order to decrease the electroporation voltage, researchers have been exploring micro devices as an effective means for achieving DNA transfection^11^,^12^. Communal electroporation on cell population have been investigated by using micro electrodes of rectangular comb^13^ or annular interdigital^14^, and microwell array-based devices^15^,^16^. Compared with population study of cell electroporation, individual cell electroporation in parallel have drawn a great attention to understand the heterogeneity and the whole cellular processes of cell transfection at the level of single cells. Although single cell electroporation has been conducted using microfluidic devices^17^, it is still immature in characterization, parallel operation, and integration with other functions.

In order to conduct an individual cell electroporation, cell positioning is required. Many method of cell positioning have been reported, for example optically-induced-dielectrophoresis (ODEP)^1^,^18^ and micro-fluidcs^17^. Dielectrophoresis (DEP) is a translational motion of a particle or cell by induced polarization in a non-uniform electric field. It is one of the most versatile methods for particle manipulation due to its label-free, favorable scaling effect, simple structure and capability to integrate with *in-situ* cellular measurements. Recent studies showed individual particles or cells could be controllably moved by modulating signal phase difference based on dielectrophoresis^19^, and the cells under DEP-based manipulation could maintain good viability^20^. Therefore, DEP-based manipulation is an effective method to move and trap cells and it is amenable for miniaturization of devices.

To comprehensively study electroporation, monitoring the dynamic process of cellular electroporation and recovery is very important. In general, the size of the electroporated pores is from 0.5 nm to 40 nm^21^, it is hard to observe the holes using optical microscope. Rapid freezing electron microscopy is used to image the holes^21^. But the real-time monitoring cannot be realized because the cells are dead before observing. Fluorescent dye, for example propidium iodide (PI) dye^22^, is a method to monitor the electroporation. The fluorescence intensity demonstrates the level of electroporation. However, this method is stained. Comparatively, electrical measurement is a simple, label-free, and practical method to monitor electroporation^23^,^24^,^25^. Furthermore, the electrical measurement can be also used to monitor exterior and interior physiological behavior of the modified cells. Patch-clamp has been used to characterize single cell electroporation^24^, but it is complicated and less efficient.

In this paper, a novel microarray chip for *in-situ*, real-time and selective electroporation on individual cells by the aid of cell positioning and impedance monitoring is proposed. The microchip consists of quadrupole-electrode units patterned into an array and pairs of planar center electrodes located at the centers of each quadrupole-electrode unit. The quadrupole-electrodes are used to move living cells onto the center electrodes based on negative dielectrophoresis (nDEP). The center electrodes are used for *in-situ* and targeted cell electroporation and monitoring the cellular dynamics in real time (e.g. the electroporation process) by measuring cellular impedance spectrum. Selective electroporation and electrical measurement on the cells in array are realized, which can achieve different electrical stimulations in parallel, while the cells are maintained in the same chip. Parallel operation can greatly optimize the stimulation parameter screening process. We present an evidence of cell electroporation by means of fluorescent dyes and show the propidium iodide (PI) can be successfully and selectively transported into HeLa cells in array. Subsequently we conduct *in-situ* impedance measurement to monitor the dynamic process of the cellular electroporation in real time. Finally, we demonstrate the use of this device to perform successful transfection of individual Hela cells with vector DNA encoding a green fluorescent.

## Results and Discussions

### Structure of the device

**Figure 1:**
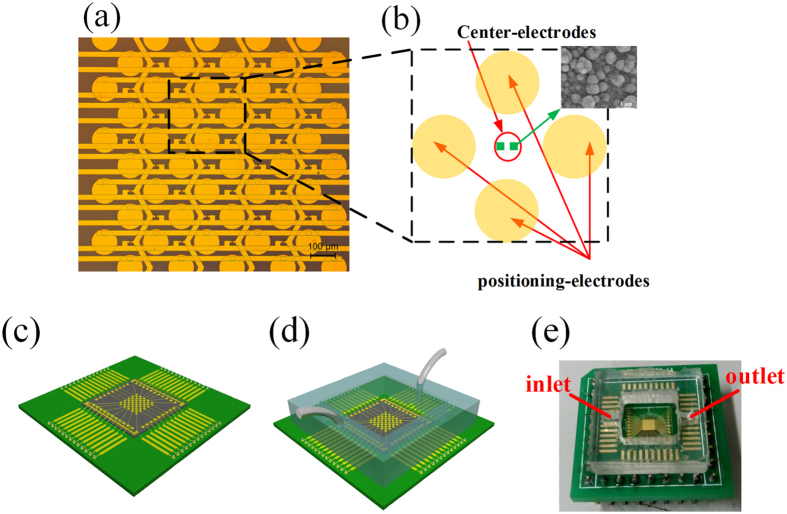
Structure of the device. (**a**) Electrode array. (**b**) Structure of one quadrupole-electrode unit with a pair of center electrodes located at the center and the inset shows the surface morphology of the center electrodes. (**c**) The chip is assembled onto the PCB. (**d**) A PDMS pool with the inlet and the outlet of the microfluidic is adhered onto the chip to form a cell sample pool. (**e**) The prototype of the device.

A complete structure of the microchip is shown in Fig. 1. The microchip consists of 4-by-8 grid of quadrupole electrode units where pairs of center electrodes are located at the centers. The area of each center electrode is 7 × 7 μm^2^ and the gap distance between two center electrodes in pair is 7 μm. To overcome the double-layer impedance existing in the electrode-electrolyte interface and enlarge the effective surface areas of the center electrodes, a surface-modification process is employed to controllably construct gold nanostructures on the electrode surfaces. ZnO nanorods are firstly grown on the center electrodes as a template, and then gold electroplating is performed on the electrodes meanwhile the ZnO nanorods are dissolved completely^26^. The surface-modified electrode surface is shown as the inset of Fig. 1b. A crossed interdigital bus-bar network is used to connect the electrodes in array with the external circuit. The fabrication of the microchip was reported in our previous paper^26^. The fabricated microchip is mounted on a printed circuit board (PCB) with wire bonding (Fig. 1c). Then a PDMS pool with inlet and outlet of microfluidic is adhered on the chip to form a cell sample pool (Fig. 1d). A syringe pump is used to control the fluid flow into the pool. Figure 1e shows the prototype of the device.

### Experimental setup

**Figure 2:**
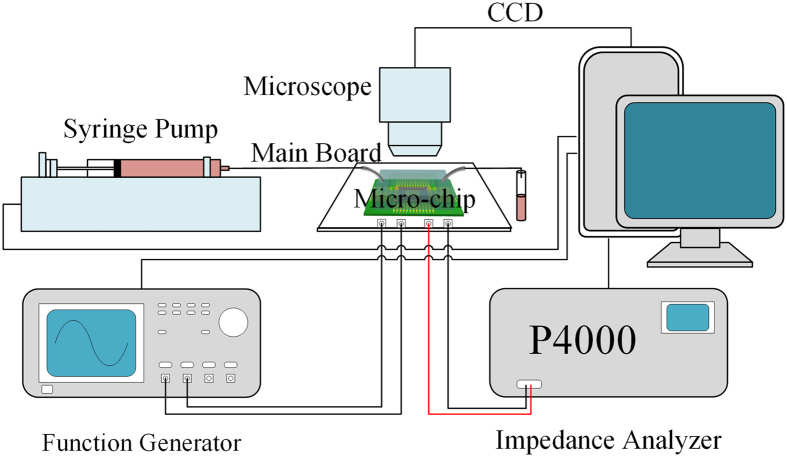
Experimental setup. It consists of a fluorescent microscope, a syringe pump that pumps sample into the pool of the device, and a main board integrating controlling circuit and I/O ports which are connected to a function generator and an impedance analyzer. The syringe pump, function generator and impedance analyzer are controlled by using a computer.

The experimental setup is depicted in Fig. 2. The system consists of a fluorescent microscope with CCD underneath which the micro-chip is mounted, a syringe pump that pumps the sample into the pool of the device, and a main board integrating controlling circuit and I/O ports of the device that are connected to a function generator and an impedance analyzer. The syringe pump, function generator and impedance analyzer are controlled by using a computer. Before experiment, the surface of the chip is coated with Poly-L-Lysine (PLL).

### Cell trap and electroporation

**Figure 3:**
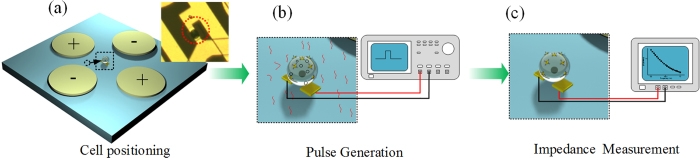
The process of cell positioning, electroporation, and impedance measurement. (**a**) Cell is positioned at the unit center under nDEP. (**b**) The electroporation is conducted onto the positioned cell using two center electrodes. (**c**) The impedance measurement is conducted to monitor the cellular dynamic.

The cell suspension (HeLa cells in culture medium) is pumped into the chip. After the cell suspension becomes stable, two alternating current (ac) sinusoidal DEP voltages with amplitude of 2.8 Vpp, frequency of 2 MHz and a phase difference of 180° generated by a function generator (AFG3250, Tektronix) are applied to the two pairs of opposite positioning electrodes while the centre electrodes are grounded for trapping and positioning the cells (Fig. 3a). Under this condition, the electric field minimum is located at the center of the DEP unit, which induces cells to be trapped therein by nDEP forces^20^. A number of experiments show that the cells suspended in culture medium can be trapped and positioned at the unit centers and form an array in less than 1 min. After the cells are positioned, the medium is exchanged to electroporation buffer by using the syringe pump at a flow rate below 25 μL/min to avoid moving the positioned cells. Afterwards, the electroporation is conducted *in situ* by applying electric pulse through the two center electrodes while the positioning electrodes are grounded as shown in Fig. 3b. After the electroporation, the impedance measurements are conducted to monitor the cellular dynamic by using an impedance analyzer (PARSTAT4000, Princeton Applied Research, USA) (Fig. 3c).

### Selective control for cell electroporation

**Figure 4:**
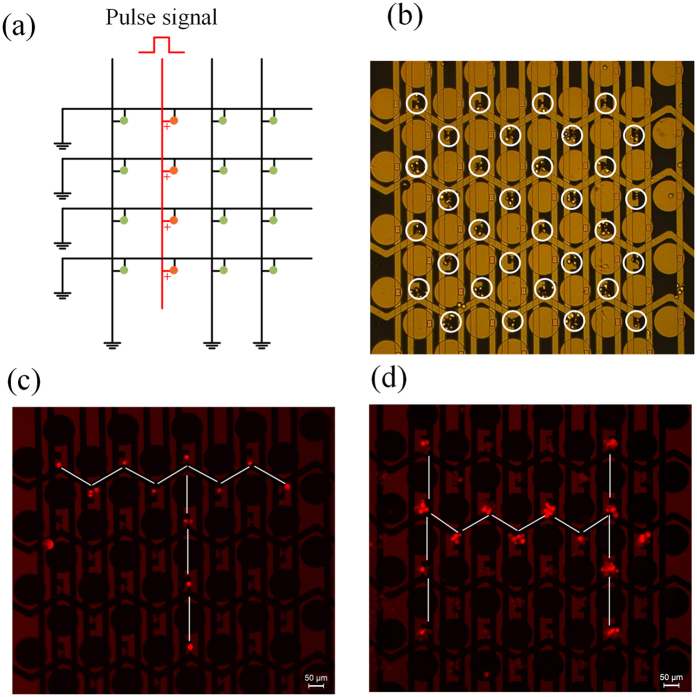
Selective control for cell electroporation. (**a**) Schematic diagram of electrical I/O connection of the center electrodes for electroporating the cells in a line of the array. (**b**) The cells are positioned on the measuring electrodes to form an array by nDEP. (**c**,**d**) Demonstrations of selective electroporation in array dyed by PI.

As mentioned above, a crossed interdigital bus-bar network with multiple longitudinal bars and lateral bars is used to connect the center electrodes with the external circuit ports. The electrical input-output (I/O) of the center electrodes in array is designed as an addressing circuit, and the electrical measurement at every unit can be accessed by addressing. For electroporation, the electrical I/O of the center electrodes in array is controlled by using a strobe circuit to realize selective cell electroporation in a specific line as shown in Fig. 4a. By this way, we can selectively conduct electroporation onto the cells prepositioned by nDEP to implement parallel operation. Figure 4b shows that the cells are positioned onto the measuring electrodes by nDEP. Two examples of selective electroporation onto the cells through use of fluorescent dyes to form like fluorescence “T” and “H” are conducted. Firstly, the living HeLa cells suspending in electroporation buffer with PI dyes are positioned onto the measuring electrodes to form an array by nDEP. Then a pulse signal with an amplitude of 14 V, a pulse width of 100 μs, a pulse number of 10, and a period of 1 s are applied to the predetermined latitudinal bar and longitudinal bar successively while other bars are grounded as shown in Fig. 4a. Finally, the cells are observed using a fluorescence microscope. The results are shown in Fig. 4c,d. Repeated experiments (more than three times) showed the efficiency of the selective electroporation is higher than 90%.

### Cell viability and electroporation analysis

**Figure 5:**
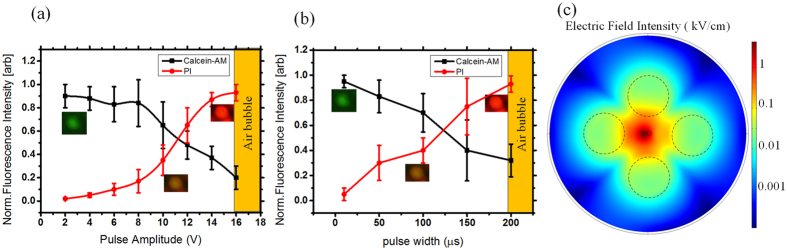
Electroporation analysis using fluorescent dyes. (**a**) HeLa cells subjected to different electroporation voltage correspond different PI and Calcein-AM fluorescence intensity. Error bars indicate statistic characteristics of fluorescence intensity in one chip. (**b**) The fluorescence intensity of PI and Calcein-AM corresponding to different pulse width of electroporation signal. (**c**) The distribution of the electric field intensity within one unit under an electroporation voltage of 10 V.

In applications of electroporation (e.g. gene transfection), good viability of the modified cell must be retained and the pores induced on the cell membrane need to be resealed. This is known as reversible electroporation. In order to assess this, we use a combination of two dyes to indicate both electroporation rate and cell viability. PI is a membrane impermeable dye that has low auto fluorescence. However, in the presence of DNA, the dye will bind to the nucleic acids and, as a result, fluoresces strongly red. The intensity of the red fluorescence evaluated by using software ImagJ indicates the electroporation rate^18^. Therefore, PI (Sigma Chem. Co., USA) dyes is added to 1 mL cell suspension (cells in electroporation buffer) at a concentration of 5 μg/mL, which is used to characterize the electroporation efficiency. Calcein-AM, initially non-fluorescent, will passively diffuse across the cell membrane. Once inside, enzymes in the cytosol break down the Calcein-AM molecule to produce a product that fluoresces green and is membrane impermeable. A cell that strongly fluoresces green in the presence of Calcein-AM has an intact membrane and the necessary enzymes to produce the fluorescent derivative. Therefore, Calcein-AM is used as an indicator that the cell is viable after cell electroporation. Firstly, the cells are positioned onto the center electrodes under nDEP manipulation, and then we conduct electroporation onto the cells by applying rectangular pulse signal with an amplitude (2 V, 4 V, 6 V, 8 V, 10 V, 12 V, 14 V and 16 V respectively under pulse width of 100 μs), a pulse width (10 μs, 50 μs, 100 μs, 150 μs, 200 μs respectively under amplitude of 10 V), a pulse number (10 pulses) and a period (1 s) to the center electrodes. Afterwards, we slowly exchange the electroporation buffer to the culture media containing 2 μmol/L Calcein-AM using the microfluidic control while keep the cells immovable. The flow rate is limited to quite low (at 25 μL/min) to avoid the positioned cells moving. After 5 minutes, the cells are observed using a fluorescence microscope. In order to indicate successful electroporation and good viability of the cells, we expect the cells fluoresce both red (excitation = 515 nm–560 nm, emission maximum = 590 nm) and green (excitation = 450 nm–490 nm, emission maximum = 515 nm). Figure 5a,b show the normalized fluorescence intensity of PI and Calcein-AM. In the normalization process, the maximum fluorescence intensity of the cell is regarded as 1 and the minimum intensity of the unstained cells is as 0. The error bars mean the standard deviation of the normalized fluorescence intensity for ten measurements.

Figure 5a indicates that at low electroporation voltage (such as 2 V, 4 V, 6 V and 8 V), the induced pores on the cytomembrane are tiny, which result in negligible red fluorescence and strong green fluorescence (indicating good viability). When the electroporation voltage is larger than 8 V, the cells exhibit strong red fluorescence while the green fluorescent intensity drops off. When the voltage achieves 14 V and above, the green fluorescent intensity is degraded to a low level indicating worse viability of the cells. When the voltage is above 16 V, electrolysis-induced air bubbles are observed. Based on these, we propose an optimal electroporation voltage range of 10–14 V. Figure 5b shows the green fluorescence is strong (indicating good viability) when the pulse width is below 100 μs. When the pulse width is larger than 150 μs, the cells exhibit strong red fluorescence while the green fluorescent intensity drops off, which indicates worse viability of cells. When the pulse width is larger than 200 μs, electrolysis-induced air bubbles are observed. Based on the above results, we propose an optimal pulse width of 100 μs.

The electrical field within one quadrupole-electrode unit of the micro-chip under the electroporation voltage of 10 V onto the center electrodes while the positioning electrodes are grounded is simulated using COMSOL AC/DC Module and is shown in Fig. 5c. The material of the model is set as a culture medium and the boundary is set as an insulator. Above 600,000 elements are meshed. The electric field intensity at the center is the highest and slightly larger than 1 kV/cm, which is feasible for the electroporation of this cell line^18^.

### Monitoring cell electroporation using impedance measurement

**Figure 6:**
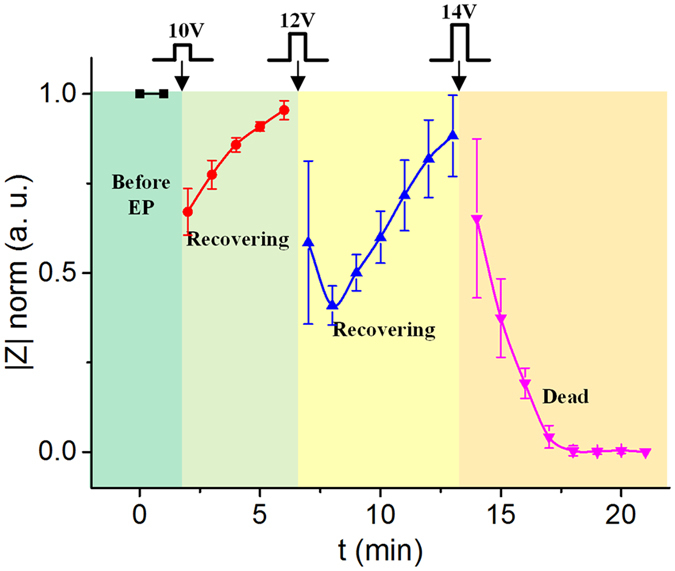
The single cellular impedances at 100 kHz before and after electroporation.

The center electrodes in the microchip can be used for not only electroporation but also impedance measurement onto the cells prepositioned on the electrodes. As mentioned above, the impedance measurement is realized by using an addressing circuit^26^. In this study, we use the impedance measurement to monitor the cellular dynamic behavior *in situ* before and after electroporation to provide an unstained and real-time approach to study electroporation. The living HeLa cells are positioned onto the center electrodes, and then the cells are incubated for about 3 hours to make them more stable. Rectangular pulse signals with different amplitudes (10 V, 12 V and 14 V), the same pulse width (100 μs), the same pulse number (10 pulses) and the same period (1 s) are applied to the center electrodes successively for executing cell electroporation. After each series of pulses, the impedance measurements are conducted on the cell in real time. The normalized impedances at 100 kHz before and after the electroporation are show in Fig. 6. The normalized impedances are calculated by regarding the living cell impedance before the electroporation as 1 and the dead cell impedance as 0. The error bars mean the standard deviation of the normalized impedance for the measurements of 3 single cells. It is seen that the cellular impedance deceases after each action of the electroporation. The electroporation induces pores on the cell membrane, which increases the permeability of the membrane and thus decreases the cellular impedance. Higher electroporation voltage corresponds to larger pores on the membrane, and therefore brings about lower impedance. In addition, we find that the impedance drops for the first two minutes after executing the electroporation under the pulse voltages of 10 V and 12 V, and then it rebound gradually, which indicates the cell is recovering. The recovering duration is about 200–300 s that agrees with other reports^22^. When the electroporation voltage is too high, for example 14 V in this experiment, the cellular impedance decreases monotonously and is not rebounded, which indicates the cell apoptotic. To further verify the result, we conduct an independent electroporation by using 14 V onto the cells. The impedance measurement shows the same change that the impedance does not rebound after the electroporation. The above experiments are repeatable, which reveals intrinsic relevance between electroporation and cellular impedance.

### Transfection of plasmid by electroporation

**Figure 7:**
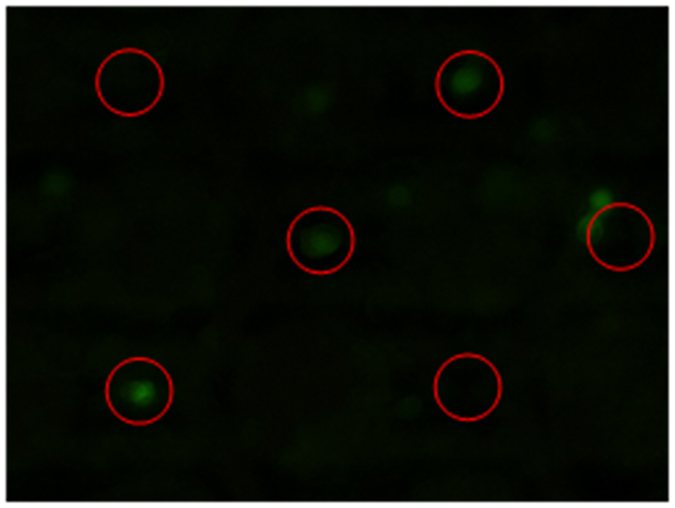
The cell transfection result. The red circles indicate sites where the center electrodes locate. The successfully transfected cells fluoresce green.

We further conducted plasmid transfection by electroporation. For plasmid transfection assays, cell suspensions are centrifuged and re-suspended to a density of 2 × 10^5^ cells mL^−1^ in the electroporation buffer containing 120 μg/mL of the pEGFP-N1 plasmid. The prepared cell suspensions are poured into the microchip, the cells are positioned at the unit centers in array under nDEP, and then the electrical pulses (12 V) are applied to the center electrodes for conducting cell electroporation. After electroporation, we slowly exchange the electroporation buffer to the culture media by using the syringe pump and then the microchip is moved into an incubator at 37 °C with a 5% CO_2_ atmosphere for cell culture. After 24 hours, fluorescence microscopic images are taken. The successfully transfected cells will emit 507 nm green fluorescence when they are excited by a 488 nm light. One of the cell transfection results is show in Fig. 7. The red circles indicate the sites where the center electrodes locate. The individual cells fluorescing green shows that the cells are successfully transfected *in situ*. It is seen some of cells are slightly deviated from the center electrodes due to cell migration and exchanging liquid.

## Conclusion

In this paper, a novel micro array chip for *in-situ* and selective electroporation of individual cell in array integrated with cell positioning and real-time impedance measurement is proposed. We present an evidence of cell electroporation using this method through use of fluorescent dyes, showing that PI can be successfully and selectively transported into HeLa cell in array. In addition, we use an impedance measurement to monitor the cell electroporation in real time offering a distinctive method to study cellular electroporation dynamics. Finally, we show the use of this device to perform successful transfection of individual HeLa cells with vector DNA encoding a green fluorescent. The developed microarray chip integrating selective *in-situ* electroporation with cell-array positioning and real-time electrical measurement has wide potential applications on such as tumor therapy, tracking cellular natural pathology conditions, and stem cell differentiation.

## Materials and Methods

### Cell culture

Human carcinoma (HeLa) cells are cultured as a monolayer in a 60 mm petri dish containing Dulbecco’s Modified Eagle Medium (DMEM) supplemented with 10% fetal bovine serum at 37 °C in a 5% CO_2_ atmosphere. The cells are harvested at the log phase of growth by 0.25% trypsin/EDTA from the petri dish, and then are resuspended in medium at a concentration of 2 × 10^5^ cells mL^−1^.

### Electroporation buffer

The electroporation buffer containing 10 mM NaCl, 1.7 mM MgCl_2_, 100 mM Sorbitol, and 10 mM HEPES (pH adjusted to 7.4 at 25 °C) are prepared and used in the electroporation.

### Device fabrication

In order to integrate all electrode arrays and crossed interdigital bus-bars in one chip, three metallic layers insulated by oxide layers are adopted. The fabrication process starts with depositing the first metallic layer comprised of titanium and gold (Ti/Au) on a glass wafer. The metallic film is patterned to form the longitudinal bars and one of the two center electrodes. Then the chip is deposited with an insulating layer of SiO_2_. The SiO_2_ layer is selectively etched to expose the center electrodes. Afterwards, the second Ti/Au layer is deposited and patterned to form the latitudinal bars and the other electrode of the center electrode pair. The second SiO_2_ layer is deposited and etched to expose the pair of center electrodes and open holes to expose the DEP electrode bars. Finally, the third Ti/Au layer is deposited and patterned to form the DEP circular electrodes as well as the pair of center electrodes^26^.

### PDMS pool fabrication

The fabrication processes of PDMS pool starts with dimethylsiloxane (PDMS) prepolymer mixture (Sylgard 184 Silicone Elastomer: Sylgard 184 Curing Agent = 10:1 by volume, degassed in vacuum for 10 minutes.) poured into a preformed mold by machining and subsequently baked/cured at 60 °C for 3 hours. Then PDMS pool is peeled off from the mold. Finally, the PDMS pool is adhered onto the chip using silica gel.

### Impedance measurement

The HeLa cell suspension is poured into the sample pool of a microchip. Under the nDEP, the cells are positioned at the center of the positioning units (where the center electrodes are located) and formed an array. Then the DEP voltages are switched off and the positioning electrodes are grounded. The center electrodes are connected to an electrochemical workstation (PARSTAT 4000, Princeton Applied Research, USA) to perform real-time impedance measurements onto the trapped cells. The electrical input-output (I/O) of the center electrodes in array is designed as an addressing circuit, and the impedance measurement at every unit can be accessed by addressing circuit^26^.

### Fluorescence measurement

When the cell is stained, fluorescence images are captured using a fluorescence microscope (DM2500, Leica, Germany). Then we use ImageJ to calculate the fluorescence intensity of cells. The maximum fluorescent intensity of a cell is regard as 1 and the minimum intensity of unstained cell is regarded as 0. Basing on this, the normalized fluorescence intensity is calculated.

## Additional Information

**How to cite this article**: Guo, X. and Zhu, R. Controllable *in-situ* cell electroporation with cell positioning and impedance monitoring using micro electrode array. *Sci. Rep*. **6**, 31392; doi: 10.1038/srep31392 (2016).

## References

[b1] Wang C. H. . Dielectrophoretically-assisted electroporation using light-activated virtual microelectrodes for multiple DNA transfection. Lab on a Chip 14, 592–601 (2014). 24322338 10.1039/c3lc51102b

[b2] Zhang X. J. & Godbey W. T. Viral vectors for gene delivery in tissue engineering. Advanced Drug Delivery Reviews 58, 515–534 (2006). 16762441 10.1016/j.addr.2006.03.006

[b3] Xiong F., Mi Z. & Gu N. Cationic liposomes as gene delivery system: transfection efficiency and new application. Pharmazie 66, 158–164 (2011). 21553643

[b4] Casteilla L. . Virus-based gene transfer approaches and adipose tissue biology. Current Gene Therapy 8, 79–87 (2008). 18393829 10.2174/156652308784049354

[b5] Jafari M., Soltani M., Naahidi S., Karunaratne D. N. & Chen P. Nonviral Approach for Targeted Nucleic Acid Delivery. Current Medicinal Chemistry 19, 197–208 (2012). 22320298 10.2174/092986712803414141

[b6] Partridge K. A. & Oreffo R. O. C. Gene delivery in bone tissue engineering: Progress and prospects using viral and nonviral strategies. Tissue Engineering 10, 295–307 (2004). 15009954 10.1089/107632704322791934

[b7] Mitragotri S. Innovation - Healing sound: the use of ultrasound in drug delivery and other therapeutic applications. Nature Reviews Drug Discovery 4, 255–260 (2005). 15738980 10.1038/nrd1662

[b8] Tao W., Wilkinson J., Stanbridge E. J. & Berns M. W. Direct Gene-Transfer into Human Cultured-Cells Facilitated by Laser Micropuncture of the Cell-Membrane. Proceedings of the National Academy of Sciences of the United States of America 84, 4180–4184 (1987). 3473500 10.1073/pnas.84.12.4180 PMC305048

[b9] Tirlapur U. K. & Konig K. Cell biology - Targeted transfection by femtosecond laser. Nature 418, 290–291 (2002). 12124612 10.1038/418290a

[b10] Satkauskas S., Ruzgys P. & Venslauskas M. S. Towards the mechanisms for efficient gene transfer into cells and tissues by means of cell electroporation. Expert Opinion on Biological Therapy 12, 275–286 (2012). 22339479 10.1517/14712598.2012.654775

[b11] Wei Z. W., Huang H., Liang Z. C. & Li Z. H. A High Performance Electroporation Chip Integrating Multi-Well Plate and Annular Interdigital Microelectrodes. *Mems 2010: 23rd Ieee International Conference on Micro Electro Mechanical Systems, Technical Digest*, 951–954 (2010).

[b12] Lin Y. C., Li M., Fan C. S. & Wu L. W. A microchip for electroporation of primary endothelial cells. Sensors and Actuators a-Physical 108, 12–19 (2003).

[b13] Olbrich M., Rebollar E., Heitz J., Frischauf I. & Romanin C. Electroporation chip for adherent cells on photochemically modified polymer surfaces. Applied Physics Letters 92 (2008).

[b14] Wei Z., Huang H., Liang Z. & Li Z. A High Performance Electroporation Chip Integrating Multi-Well Plate and Annular Interdigital Microelectrodes. *2010 IEEE 23rd International Conference on Micro Electro Mechanical Systems*,Hong Kong. (24 Jan–28 Jan 2010).

[b15] Jain T., McBride R., Head S. & Saez E. Highly parallel introduction of nucleic acids into mammalian cells grown in microwell arrays. Lab on a Chip 9, 3557–3566 (2009). 20024036 10.1039/b913794g PMC3033197

[b16] Jain T., Papas A., Jadhav A., McBride R. & Saez E. *In situ* electroporation of surface-bound siRNAs in microwell arrays. Lab on a Chip 12, 939–947 (2012). 22245984 10.1039/c2lc20931d PMC3392120

[b17] Valero A. . Gene transfer and protein dynamics in stem cells using single cell electroporation in a microfluidic device. Lab on a Chip 8, 62–67 (2008). 18094762 10.1039/b713420g

[b18] Valley J. K. . Parallel single-cell light-induced electroporation and dielectrophoretic manipulation. Lab on a Chip 9, 1714–1720 (2009). 19495455 10.1039/b821678a PMC2752467

[b19] Guo X. L. & Zhu R. Controllably moving individual living cell in an array by modulating signal phase difference based on dielectrophoresis. Biosensors & Bioelectronics 68, 529–535 (2015). 25638795 10.1016/j.bios.2015.01.052

[b20] Guo X. L. & Zhu R. A biocompatible microchip and methodology for efficiently trapping and positioning living cells into array based on negative dielectrophoresis. Journal of Applied Physics 117 (2015).

[b21] Chang D. C. & Reese T. S. Changes in Membrane-Structure Induced by Electroporation as Revealed by Rapid-Freezing Electron-Microscopy. Biophysical Journal 58, 1–12 (1990). 2383626 10.1016/S0006-3495(90)82348-1 PMC1280935

[b22] Chen S. C. . Delivery of molecules into cells using localized single cell electroporation on ITO micro-electrode based transparent chip. Biomedical Microdevices 14, 811–817 (2012). 22674171 10.1007/s10544-012-9660-9

[b23] DeBruin K. A. & Krassowska W. Modeling electroporation in a single cell. I. Effects of field strength and rest potential. Biophysical Journal 77, 1213–1224 (1999). 10465736 10.1016/S0006-3495(99)76973-0 PMC1300413

[b24] Ryttsen F. . Characterization of single-cell electroporation by using patch-clamp and fluorescence microscopy. Biophysical Journal 79, 1993–2001 (2000). 11023903 10.1016/S0006-3495(00)76447-2 PMC1301089

[b25] Khine M., Lau A., Ionescu-Zanetti C., Seo J. & Lee L. P. A single cell electroporation chip. Lab on a Chip 5, 38–43 (2005). 15616738 10.1039/b408352k

[b26] Guo X. L., Zhu R. & Zong X. L. A microchip integrating cell array positioning with *in situ* single-cell impedance measurement. Analyst 140, 6571–6578 (2015). 26282920 10.1039/c5an01193k

